# Integrative proteomic characterization of trace FFPE samples in early-stage gastrointestinal cancer

**DOI:** 10.1186/s12953-022-00188-0

**Published:** 2022-04-09

**Authors:** Lingling Li, Hui Liu, Yan Li, Chunmei Guo, Bing Wang, Dan Shen, Qiao Zhang, Chen Ding

**Affiliations:** 1 State Key Laboratory of Genetic Engineering and Collaborative Innovation Center for Genetics and Development, School of Life Sciences, Institute of Biomedical Sciences, Human Phenome Institute, Fudan University, Shanghai, 200433 China; 2 State Key Laboratory Cell Differentiation and Regulation, Overseas Expertise Introduction Center for Discipline Innovation of Pulmonary Fibrosis, (111 Project), College of Life Science, Henan Normal University, Xinxiang, 453007 Henan China; 3 Academy of Medical Science, Zhengzhou University, Zhengzhou, 450052 China

**Keywords:** Early-stage gastrointestinal cancer, Proteomics, 10,000 phosphosites, Immune infiltration, Kinases characterization

## Abstract

**Background:**

The surveillance and therapy of early-stage cancer would be better for patients’ prognosis. However, the extreme trace amount of tissue samples in different stages have limited in portraying the characterization of early-stage cancer. Therefore, we focused on and presented comprehensive proteomic and phosphoproproteomic profiling of the trace FFPE samples from early-stage gastrointestinal cancer, and then explored the potential biomarkers of early-stage gastrointestinal cancer.

**Methods:**

In this study, a quantitative proteomic method with chromatography with mass spectrometry (LC-MS/MS) was used to analyse the proteomic difference between the trace early-stage esophageal squamous cell carcinoma (EESCC) and early-stage duodenum adenocarcinoma cancer (EDAC).

**Results:**

We identified ~ 6000 proteins and > 10,000 phosphosites in single trace FFPE samples. Comparative analysis disclosed the diverse proteomic features of tumor tissues compared with paired normal tissue of EESCC and EDAC, and revealed the difference of EESCC and EDAC was derived from their origin normal tissue. The distinct separation of EESCC and EDAC illustrated the functions of cell cycle (RB1 T373, EGFR T693) in EESCC, and the positive impacts of apoptosis, metabolic processes (MTOR and MTOR S1261) in EDAC. Furthermore, we deconvoluted the immune infiltration of early-stage gastrointestinal cancer, in which higher immune cell signatures were detected in EDAC, and showed the specific cytokines in EESCC and EDAC. We performed kinases-substates relationship analysis and elucidated the specific proteomic kinase characterization of EESCC and EDAC, and proposed the medicative effects and corresponding drugs for EESCC and EDAC at the clinic.

**Conclusion:**

We disclosed the specific immune characterization of the early-stage gastrointestinal cancer, and presented potential makers of EESCC (EGFR, PDGFRB, CDK4, WEE1) and EDAC (MTOR, MAP2K1, MAPK3). This study represents a major stepping stone towards investigating the carcinogenesis mechanism of gastrointestinal cancer, and providing a rich resource for medicative strategy in the clinic.

**Graphical Abstract:**

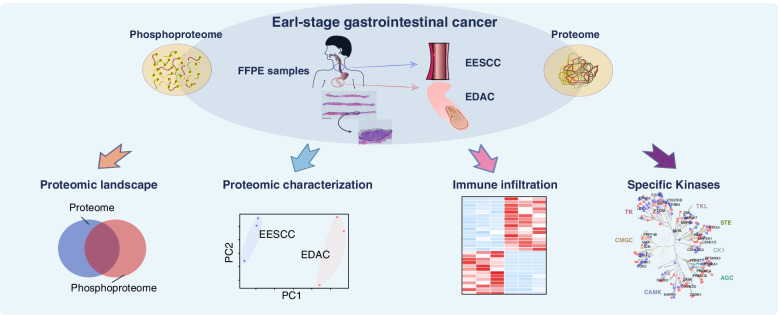

**Supplementary Information:**

The online version contains supplementary material available at 10.1186/s12953-022-00188-0.

## Introduction

Cancer is still a major health problem worldwide, leading nearly 10 million deaths every year [1, 2]. Surgery is the predominant curative treatment strategy in advanced stages (T2 to T4 stages), with poor quality of life (QOL) and low five-year survival rate (< 30%) [3]. Early screening and diagnosis of disease, approved by World Health Organization (WHO) has been prominent nowadays, especially in gastrointestinal cancer. In addition, advances of endoscopic submucosal dissection (ESD) [4] have achieved the early detection cancers (T1 stage), especially in gastrointestinal cancer, with higher QOL and significantly improved overall survival rate (> 90%) [5–7]. The major events in advanced-stage cancer, appeared to be identified in as early as in early-stage cancer, thus, the surveillance and therapy of early-stage cancer would be better for patients’ prognosis. However, the extreme trace amount of tissue samples in different stages have limited in portraying the characterization of early-stage cancer.

Pathologically, formalin-fixed, paraffin-embedded (FFPE) biospecimens represent gold standard for archiving pathology samples, keeping the tissues stability, providing a valuable resource for clinical and biomarker researches [8]. In addition, FFPE tissue biopsies showed a high degree of the proteome pattern similarity between histological regions samples collected for 1 and 15 years, and facilitates tumor stratification [9]. During the last decade, great progress has been made in mass spectrometry-based proteomics, providing chances for employing biobanked FFPE samples to many types of cancers to reveal cancer proteomic characterization. Specifically, proteomic analysis of colon rectal cancer (CRC) revealed decreased T cell infiltration and increased glycolysis in CRC [10]; *Sai Ge* et.al, described a proteomic landscape of diffuse-type gastric cancer, and illustrated the overrepresentation of immune response in the third subtypes with worst survival [11]. However, the proteomic profiling of trace FFPE samples in early-stage cancer remain largely unknow.

Here, we collected FFPE samples of early-stage gastrointestinal cancer, including early-stage esophageal squamous cell carcinoma (EESCC) and early-stage duodenum adenocarcinoma cancer (EDAC). We presented a comprehensive proteomic landscape of early-stage gastrointestinal cancer, with the identification of ~ 6000 proteins and > 10,000 phosphosites in single trace FFPE samples. We revealed the functional classification and proteomic characterization of EESCC and EDAC. In addition, we elucidated the immune infiltration, cytokines types, specific kinases of EESCC and EDAC, and proposed the potential kinase clinic strategy for EESCC and EDAC, providing a novel useful resource for potential therapeutic approaches for gastrointestinal cancer.

## Materials and methods

### Processing of formalin-fixed, paraffin-embedded (FFPE) specimens

In this study, all the tissue samples from the corresponding substages were separately dissected from the formalin-fixed, paraffin-embedded (FFPE) slides, and were prepared and provided by Zhongshan Hospital, Fudan University. The samples of EESCC and EDAC were derived from different stage showing as follows (Table 1).

**Table 1:** The pathological information of tumor tissue samples in EESCC and EDAC

Samples	Stage
EESCC_T1	Muscularis mucosa stage
EESCC_T2	Muscularis mucosa stage
EESCC_T3	Submucosa stage
EDAC_T1	Low-grade intraepithelial neoplasia
EDAC_T2	Low-grade intraepithelial neoplasia
EDAC_T3	High-grade intraepithelial neoplasia

The study was carried out in compliance with the ethical standards of Helsinki Declaration II and approved by the Institution Review Board of Fudan University Zhongshan Hospital (B2019-200R). For clinical sample preparation, slides (10 μm thick) from FFPE blocks were macro-dissected, deparaffinized with xylene and washed with ethanol. All the selected specimens were evaluated and confirmed by two or three experienced and board-certified gastrointestinal pathologists, and materials were aliquoted and kept in storage at − 80 °C until further processing.

### Protein extraction and digestion

Nearly 50 μL TCEP buffer (2% deoxycholic acid sodium salt, 40 mM 2-chloroacetamide, 100 mM tris-phosphine hydrochloride, 10 mM (2-carboxyl)-phosphine hydrochloride, 1 mM phenylmethylsulfonyl fluoride mixed with MS water, PH 8.5) was added into 1.5 mL EP tubes with prepared samples (0.01 cm × 0.01 cm), and then heated in a 99 °C metal bath for 30 min. Cool to room temperature, 3 μg trypsin (REF: V528A, PROMEGA) was added into each tube and digested for 18 h in a 37 °C incubator. Then, 13 μL 10% formic acid was added into each tube and made vortex for 3 min, and then sedimentation for 5 min (12,000 g). After that, a new 1.5 mL tube with 350 μL buffer (0.1% formic acid in 50% acetonitrile) is needed for collected the supernatant for extraction (vortex for 3 min, and then 12,000 g sedimentation for 5 min). And then the supernatant was transferred into a new tube for drying in 60 °C vacuum drier. After drying, 100 μL 0.1% formic acid was needed for dissolving the peptides and vortex for 3 min, and then sedimentation for 3 min (12,000 g). The supernatant was picked into new tube and then desalinated. Before desalination, the activation of pillars with 2 slides of 3 M C8 disk is required, and the lipid is as follows: 90 μL 100% acetonitrile twice, 90 μL 50 and 80% acetonitriler once in turn, and then 90 μL 50% acetonitrile once. After pillar balance with 90 μL 0.1% formic acid twice, the supernatant of the tubes was loading into the pillar twice, and decontamination with 90 μL 0.1% formic acid twice. Lastly, 90 μL elution buffer (0.1% formic acid in 50% acetonitrile) was added into the pillar fir elution twice and only the effluent was collected for MS. And then the collect of lipid was put in 60 °C vacuum drier for drying.

### Proteome analysis in LC-MS/MS analysis

For the proteomic profiling of samples, peptides were analyzed on a Q Exactive HF-X Hybrid Quadrupole-Orbitrap Mass Spectrometer (Thermo Fisher Scientific, Rockford, IL, USA) coupled with a high-performance liquid chromatography system (EASY nLC 1200, Thermo Fisher). Dried peptide samples re-dissolved in Solvent A (0.1% FA in water) were loaded to a 2-cm self-packed trap column (100-μm inner diameter, 3 μm ReproSil-Pur C18-AQ beads, Dr. Maisch GmbH) using Solvent A and separated on a 150-μm-inner-diameter column with a length of 30 cm (1.9 μm ReproSil-Pur C18-AQ beads, Dr. Maisch GmbH) over a 150 min (EESCC and EDAC). The eluted peptides were ionized under 2.0 kV and introduced into mass spectrometer). MS was performed under a data-dependent acquisition mode. For the MS1 Spectra full scan, ions with m/z ranging from 300 to 1400 were acquired by Orbitrap mass analyzer at a high resolution of 120,000. The automatic gain control (AGC) target value was set as 3E6. The maximal ion injection time was 80 ms. MS2 Spectra acquisition was performed in the ion trap mode at a rapid speed. Precursor ions were selected and fragmented with higher energy collision dissociation (HCD) with a normalized collision energy of 27%. Fragment ions were analyzed by the ion trap mass analyzer with the AGC target at 5E4. The maximal ion injection time of MS2 was 20 ms. Peptides that triggered MS/MS scans were dynamically excluded from further MS/MS scans for 12 s.

### Proteomics data processing and analyzing

All profiling data qualified were downloaded from firmiana platform (https://phenomics.fudan.edu.cn/firmiana/) against the human RefSeq protein database (updated on 04-07-2013) in the National Center for Biotechnology Information (NCBI). Student’s t test was used for statistical analysis, and data from more than two groups were analyzed by one-way ANOVA in SPSS Statistics 19.0 and a subsequent Fisher’s least significant difference t test. Results were considered significantly when *p* < 0.05.

The maximum number of missed cleavages was set to 2. A mass tolerance of 20 ppm for precursor and 0.5 Da for production was allowed. The fixed modification was cysteine carbamidomethylation and the variable modifications were N-acetylation and oxidation of methionine. For the quality control of proteins identification, the target-decoy based strategy was applied to confirm the FDR (False Discovery Rate) of both peptide and protein was lower than 1%. Percolator was used to obtain the probability value (q-value), validating the FDR (measured by the decoy hits) of every peptide-spectrum match (PSM) was lower than 1%. Then all the peptides shorter than seven amino acids were removed. The cutoff ion score for peptide identification was 20. All the PSMs in all fractions were combined for protein quality control, which was a stringent quality control strategy. The q-values of both target and decoy peptide sequences were dynamically increased until the corresponding protein FDR was less than 1% employing the parsimony principle. Finally, to reduce the false positive rate, the proteins with at least one unique peptide were selected for further investigation.

### Phosphopeptide enrichment and analysis

The phosphoproteome samples were prepared by Fe-NTA Phosphopeptide Enrichment Kit (Thermo, Catalog: A32992) according to the manufacturer’s instruction. The peptides were resuspended in 200 μL binding/wash buffer and loaded to the equilibrated spin column. The resin was mixed with the sample by gently tapping. The mixture was incubated for 30 min and centrifuged at 1000×g for 30 s to discard the flowthrough. The column was then washed by 200 μL of binding/wash buffer and centrifuged at 1000×g for 30 s for 3 times and washed by 200 μL of LC-MS grade water for one additional time. The phosphopeptide was eluted by adding 100 μL of elution buffer and centrifuged at 1000×g for 30 s for 2 times. Phosphopeptides were dried down for LC-MS/MS analysis.

For the phosphoproteomic analysis, the phosphopeptides were analyzed on Orbitrap Fusion Lumos Tribrid Mass Spectrometer (Thermo Fisher Scientific, Rockford, IL, USA) equipped with an Easy nLC-1000 (Thermo Fisher Scientific, Rockford, IL, USA) and a Nanoflex source (Thermo Fisher Scientific, Rockford, IL, USA). Dried peptide samples re-dissolved in Solvent A (0.1% FA in water) were loaded to a 2-cm self-packed trap column using Solvent A and separated on a 150-μm-inner-diameter column with a length of 30 cm over a 150 min gradient (buffer A: 0.1% FA in water; buffer B: 0.1% FA in 80% ACN) at a constant flow rate of 600 nL/min (0-150 min, 0 min, 4% B; 0-10 min, 4-15% B; 10-125 min, 15-30% B; 125-140 min, 30-50% B; 140-141 min, 50-100% B; 141-150 min, 100% B). The eluted phosphopeptides were ionized and detected. Mass spectra were acquired over the scan range of m/z 350-1500 at a resolution of 120,000 (AUG target value of 5E5 and max injection time 50 ms). For the MS2 scan, the higher-energy collision dissociation fragmentation was performed at a normalized collision energy of 30%. The MS2 AGC target was set to 1E4 with a maximum injection time of 10 ms, Peptide mode was selected for monoisotopic precursor scan, and charge state screening was enabled to reject unassigned 1+, 7+, 8+, and > 8+ ions with a dynamic exclusion time of 45 s to discriminate against previously analyzed ions between ±10 ppm.

### Principal component analysis (PCA) of trace FFPE samples

We performed PCA on a total of proteins/phosphoproteins identified in 6 paired gastrointestinal cancer samples to illustrated the global proteomic difference between EESCC and EDAC. The PCA function under the scikit-learn R package was implemented for unsupervised clustering analysis with the parameter ‘n_components = 2’ on the expression matrix of global proteomic data. A colored ellipse represented the 95% confidence coverage for each group.

### Biological pathways enrichment analysis

To investigate the dominant signaling pathways of the overlap proteins and each concentrated gradient of trace samples, we used gens sets of molecular pathways in DAVID [12]. For this analysis, pathways from the GOBP/KEGG database were considered. Statistical significance was considered when *P* value was less than 0.05 and FDR *q* value was no more than 0.1.

### Kinase activity prediction and Phosphopeptide analysis

The phospho-proteome data of 6 samples were searched against the same database with MaxQuant. The phosphorylation of S or T or Y was set as variable modification, in which three mis-cleavages were allowed, with a minimum Andromeda score of 40 for spectra matches. The ratios of identified phosphorylation sites of all samples were used to estimate the kinase activities by Kinase-Substrate Enrichment Analysis (KSEA) algorithm [13]. The information of kinase-substrate relationships was obtained from publicly available databases, including PhosphoSite [14], Phospho.ELM [15] and PhosphoPOINT [16]. The information of substrate motifs was obtained either from the literature [17] or from an analysis of the KSEA dataset with Motif (sP) [18]. The kinase-substrate-motif network analysis was referenced from PhosphoSitePlus (PSP, https://www.phosphosite.org/homeAction) [19]. Statistical analysis was performed in R (version 3.5.1) with Kruskal-Wallis test.

## Results

### Overview of proteomic landscape of trace FFPE samples in EESCC and EDAC

**Fig. 1:**
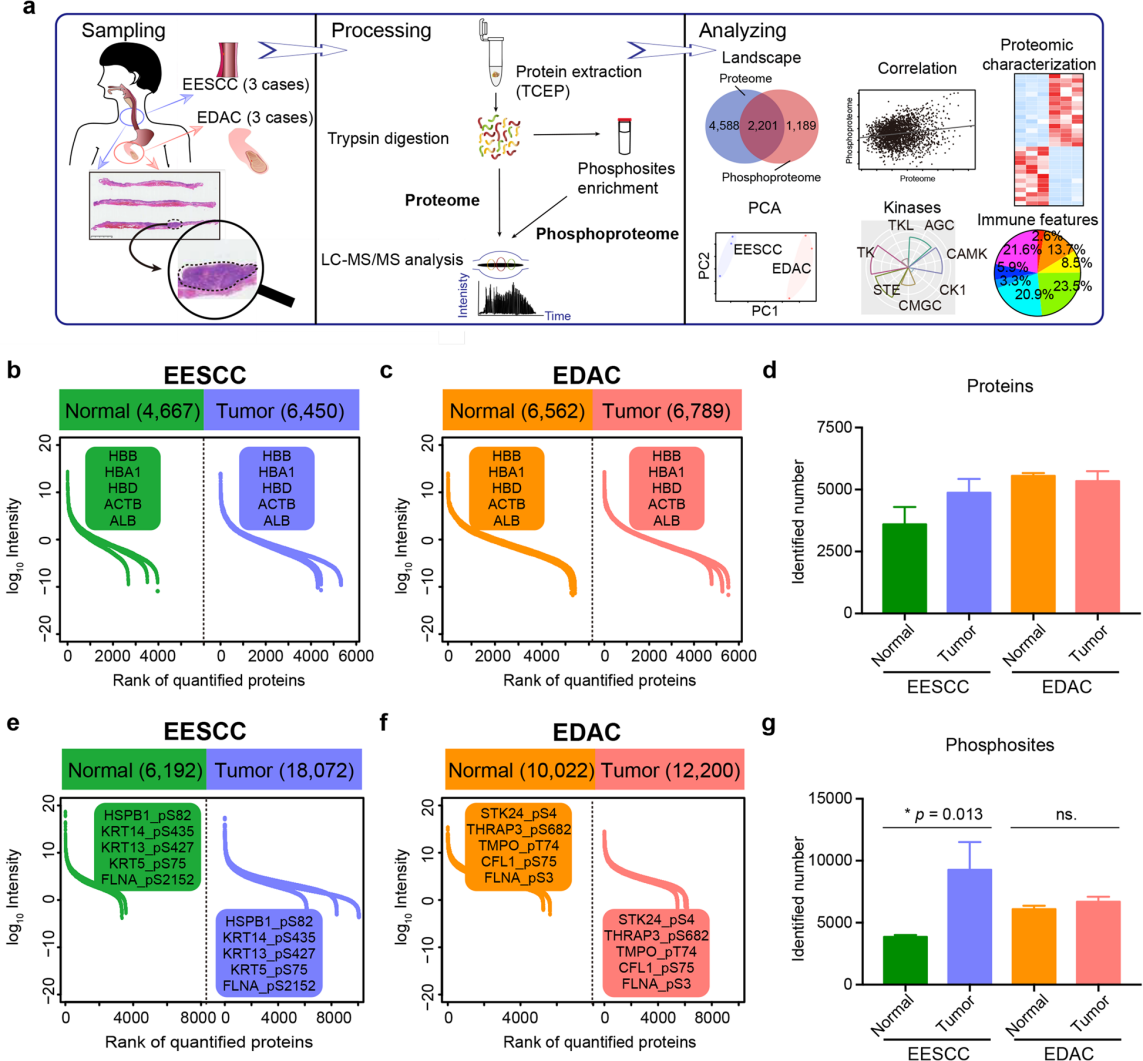
Proteomic landscape of trace FFPE samples in EESCC and EDAC. **a** Overview of the experimental design. A total of 12 samples were collected from EESCC (3 cases) and EDAC (3 cases). **b** Proteomic landscape of EESCC. **c** Proteomic landscape of EDAC. **d** The number of identified proteins in EESCC (purple) and EDAC (red). **e** Phosphoproteomic profiles of EESCC. **f** Phosphoproteomic profiles of EDAC. The high abundance proteins/phosphoproteins were shown in the box. **g** The number of identified phosphosites in EESCC and EDAC (t-test)

To characterize the comprehensive proteomic landscape of trace FFPE samples, we collected proteomics and phosphoproteomics data from 3 early-stage ESCC (EESCC) patients and 3 early-stage DC (EDAC) patients who had not experienced prior chemotherapy or radiotherapy. A schematic of the experimental design is shown in Fig. 1a and Supplementary Fig. S1a. Proteomic analysis was performed on the basis of mass spectrometry (MS)-based label-free quantification strategy [11, 20]. Protein abundance of all samples was firstly calculated by intensity-based absolute quantification (iBAQ) [21, 22] and then normalized as a fraction of the total (FOT), allowing for comparisons between experiments.

At the protein level, identification of ~ 6000 proteins were observed in single trace FFPE sample. A total of 4667 and 6450 were identified in the normal and tumor tissues of EESCC, respectively (Supplementary Fig. S1b – d). In EDAC, 6562 and 6789 proteins were detected in the normal and tumor tissues of EDAC, respectively. Interestingly, the high abundance identifies were consistent both in the tumor and normal tissues in EESCC and EDAC at the protein level, including HBB, HBA1, HBD, ACTB, ALB, etc. (Fig. 1b – d; Supplementary Table S1). Comparably, those high abundance proteins were also expressed in the human stomach cancer [23] and lung adenocarcinoma [24] and their corresponding normal tissues. These findings implied that those proteins might be highly expressed in all human tissues. Additionally, ESCC biomarkers identified in previous study, such as ACTA2, ANXA1 [25], HSPA9, THBS1 [26] etc. were also detected in EESCC, indicating the key events in advanced-stage cancer happened as earlier as in the early-stage cancer.

At the phosphoprotein level, identification of over 10,000 phosphosites was observed in the single trace FFPE sample. A total of 18,072 phosphosites corresponding to 4173 phosphoproteins, and 12,200 phosphosites corresponding to 3390 phosphoproteins were identified in tumor tissues of EESCC and EDAC, respectively (Fig. 1e – g). In addition, the highly phosphorylation (e.g., HSPB1 S82, KRT14, KRT13 S427, etc.) were both detected in tumor tissues and their paired normal tissues in EESCC (Fig. 1e; Supplementary Table S2). Whereas, the highly phosphorylation in EDAC were STK24 S4, THRAP3 S682, TMPO T74, etc. at the phosphoprotein level (Fig. 1f). Observation of significantly more phosphosites were detected in tumor tissues compared with paired normal tissues in EESCC (t test, *p* = 0.013), which was not notable in EDAC (Fig. 1g). These findings indicated the similarly high abundance identifies in EESCC and EDAC at the protein level, whereas the highly expressed phosphoproteins were different. Overall, we established a comprehensive landscape of trace FFPE samples in early-stage gastrointestinal cancer (EESCC and EDAC) at multi-omics levels, and identified ~ 6000 proteins and > 10,000 phosphosites in single FFPE samples.

### Proteomic characterization of tumor tissues compared with paired normal tissues

**Fig. 2:**
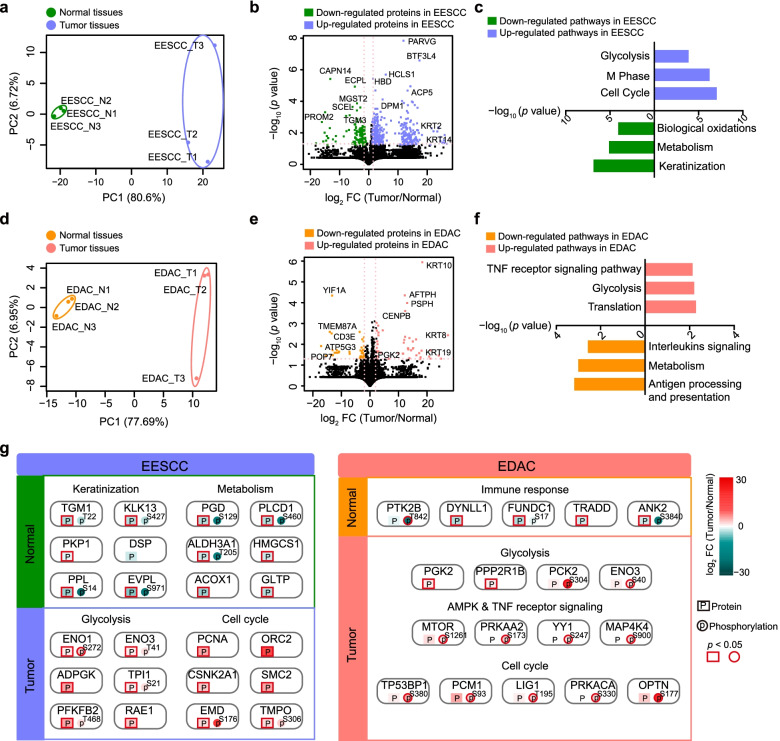
The proteomic features of tumor tissues compared with paired normal tissues in in EESCC and EDAC. **a** PCA analysis showing distinct separation between the tumor tissues and paired normal tissues in EESCC at the protein level. **b** Volcano analysis depicted the differential expressed proteins of the tumor tissues and paired normal tissues in EESCC at the protein level. **c** Bar chart presenting the functional pathways in up-regulated and down-regulated pathways in EESCC at the protein level. **d** PCA analysis showing distinct separation between the tumor tissues and paired normal tissues in EDAC at the protein level. **e** Volcano analysis depicted the differential expressed proteins of the tumor tissues and paired normal tissues in EDAC at the protein level. **f** Bar chart presenting the functional pathways in up-regulated and down-regulated pathways in EDAC at the protein level. **g** A brief summary descripting the differential proteomic features of tumor tissues and paired normal tissues in EECC (left) and EDAC (right)

To investigate the differential proteomic features of tumor tissues compared with paired normal tissues, we performed principal component analysis (PCA) at the protein and phosphoprotein levels. To an end, we found obvious separation between normal tissues and tumor tissues of EESCC at the protein level and phosphoprotein levels, as well as in EDAC (Fig. 2a, d, and Supplementary Fig. S2a, d). We then integrated the differential expressed proteins and phosphoproteins (DEPs) between the tumor tissues and paired normal tissues in EESCC and EDAC, respectively. In EESCC, we found the normal tissues highly expressed proteins/phosphoproteins participated in the primary physiological functions of the esophagus, such as keratinization (e.g., TGM1, KLK13, etc.) and metabolism (e.g., PGD, PLCD1, etc.), etc. (Fig. 2b, c, g, and Supplementary Fig. S2b, c). Over-representation of glycolysis and cell cycle were observed in the tumor tissues of EESSCC.

In EDAC, immune response (e.g., PTK2B, TRADD, etc.) was prominent in the normal tissues, including interleukins signaling, antigen processing and presentation (Fig. 2e, f, g, and Supplementary Fig. S2e, f). AMPK and TNF receptor signaling (e.g., MTOR, MAP4K1) were overrepresented in the tumor tissues of EDAC at the protein and phosphoprotein levels. As well as in EESCC, cell cycle (e.g., PCM1, LIG1, etc.) and glycolysis (e.g., PGK2, ENO3, etc.) were predominant in the tumor tissues of EDAC. Clinical Proteomic Tumor Analysis Consortium (CPTAC) show that glycolysis can be a potential target to overcome the resistance of microsatellite instability-high (MSI-H) tumors to immune checkpoint blockade [10]. These findings suggested that MSI-H might be prevalent in the gastrointestinal cancer, and glycolysis could be a potential target in EESCC and EDAC. Taken together, we disclosed the proteomic features of tumors compared with paired normal tissues in EESCC and EDAC.

### Proteomic characterization of EESCC and EDAC

To investigate the correlations between the proteome and phosphoproteome of EESCC and EDAC, we performed integration analysis and found significant association between proteome (*n* = 6450) and phosphoproteome (*n* = 4173) in EESCC (Pearson correlation, R = 0.25, *p* < 2.2E-16) (Supplementary Fig. S3a). As well, notably association between proteome (*n* = 6789) and phosphoproteome (*n* = 3390) in EDAC (Pearson correlation, R = 0.19, *p* = 8.1E-15) (Supplementary Fig. S3b), which allowed us to further investigating the characterizations of early-stage cancer.

**Fig. 3:**
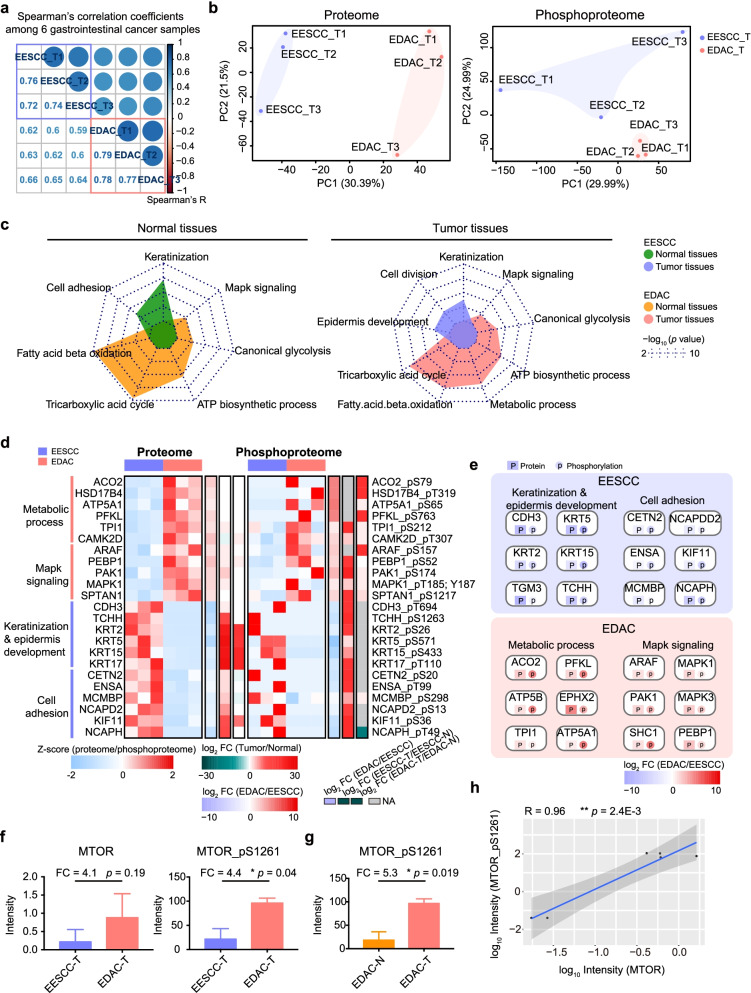
Proteomic characterization of EESCC and EDAC. **a** Spearman’s correlation coefficients among 6 gastrointestinal cancer samples. **b** PCA analysis showing distinct separation between EESCC and EDAC at the protein (left) and phosphoprotein (right) levels. **c** Comparative analysis the dominant pathways of normal tissues (top) and tumor tissues (bottom) in EESCC and EDAC. **d** Proteins in functional pathways that were differentially expressed in EESCC and EDAC at protein and phosphoprotein levels. **e** A brief of the differential proteins and functional pathways in EESCC (top) and EDAC (bottom). **f** Boxplot showing MTOR was highly expressed in EDAC at the protein (left) and phosphoprotein (right) levels (t-test). **g** Boxplot showing MTOR (S1261) was highly expressed in the tumor tissues compared with paired normal tissues (t-test). **h** Pearson’s correlation coefficients indicated significantly positive association between MTOR proteome and phosphoproteome (S1261)

Spearman’s correlation showed lower coefficients (mean = 0.62) between EESCC and EDAC than the same cancer type (mean = 0.74 (ESCC) and 0.78 (EDAC)) indicated the difference in gastrointestinal cancer (Fig. 3a). To explore the difference between EESCC and EDAC, we performed PCA at the protein and phosphoprotein levels. Visualization of PCA differentiated the proteome profiles between EESCC and EDAC, as well as at the phosphoprotein level (Fig. 3b). Interestingly, notable separation was also observed in the normal tissues between EESCC and EDAC at the protein and phosphoprotein levels (Supplementary Fig. S3c). The distinct separation suggested the fundamental difference between EESCC and EDAC.

To access whether the differential features of the tumor tissues were derived from the origins of their normal tissues, we compared the proteomes of normal tissues of EESCC and EDAC. Notably, the dominant pathways of normal tissues in the EESCC were enriched in keratinization and cell adhesion, which were also over-represented in the tumor tissues of EESCC (Fig. 3c). In the EDAC, AMPK signaling, canonical glycolysis, ATP biosynthetic process, tricarboxylic acid (TCA) cycle, and fatty acid beta oxidation, were dominant in the normal tissues and tumor tissues. These findings suggesting the diversity in the EESCC and EDAC was derived from their origin normal tissues.

The significance analysis of microarray (SAM) [27] was performed to investigate the characteristics of EESCC and EDAC at the protein level, which identified 791 differentially expressed proteins (DEPs) between EESCC and EDAC (t-test, *p* < 0.05, fold change (FC) (EDAC/EESCC) ≥ 2 or ≤ 0.5), including 678 elevated (EDAC-proteins) and 113 descend proteins (EESCC-proteins) (Supplementary Fig. S3d). GO-enrichment analysis presented that the EESCC-proteins were related to the primary functions of esophagus tissues, including keratinization (*p* = 2.0E-4) (e.g., CDH1, KRT2, etc.), cell division (*p* = 1.2E-3) (e.g., NCAPD2, MCMBP, etc.), and epidermis development (*p* = 1.7E-3) (e.g., TCHH, CETN2, etc.) (Fig. 3d, e). The EDAC-proteins participated in Mapk signaling (*p* = 7.7E-3) (e.g., MAPK1, MAPK3, etc.) and metabolic processes (*p* = 2.0E-4) (e.g., ACO2, PAK1, etc.), covering tricarboxylic acid cycle (TCA) (*p* = 2.6E-9) (e.g., SDHA, IDH1, etc.), fatty acid-beta-oxidation (*p* = 3.6E-8) (e.g., ACOX2, CPT2, etc.), ATP biosynthesis process (*p* = 6.5E-6) (e.g., ATP5B, ATP5S, etc.), canonical glycolysis (*p* = 2.8E-4) (e.g., PFKL, TPI1, etc.).

Observation of the difference between EESCC and EDAC was also identified at the phosphoprotein level. For example, the phosphorylation of keratinization−/epidermis development−/cell division- related phosphoproteins were detected in EESCC, such as KRT2 S26, KRT5 S571, CDH3 T694, TGM3 S471, NCAPD2 S13, MCMBP S298, etc. In addition, the phosphorylation of MAPK T185/Y187, MAPK3 T202, ACO2 S79, ATP5B T140, PFKL S763, etc. was elevated in EDAC (Supplementary Fig. 3e, f). Notably, the high phosphorylation was also observed in the tumor tissues compared paired normal tissues of EESCC and EDAC. Furthermore, we found the EESCC-phosphoproteins were associated in cell cycle (*p* = 1.9E-3) (e.g., RB1 T373, RBL2 S662, etc.) and signal transduction (*p* = 1.3E-3) (e.g., EGFR T693, ROCK1 S1108, etc.). The mutation of *RB1* was prevalent in ESCC [28], and the mutation of *EGFR* was gefitinib-sensitizing mutation in ESCC [29]. In our datasets, we found the significant association between RB1 T373 and EGFR T693 (Pearson correlation, R = 0.97, *p* = 1.7E-3) at the phosphoprotein level, indicating the co-functions in RB1 and EGFR in esophageal carcinogenesis (Supplementary Fig. S3g). The EDAC-phosphoproteins were involved in regulation of apoptosis (*p* = 9.9E-4) (e.g., APC S2093, ATM T1885, etc.). The mutation of *MTOR* was the driven mutation for small-bowel carcinoma, and is regarded as the potential targets in the clinic [30, 31]. In this study, we found the higher expression of MTOR (t-test, FC (EDAC/EESCC) = 4.1, *p* = 0.19) and MTOR S1261 (t-test, FC (EDAC/EESCC) = 4.4, *p* = 0.04) in EDAC (Fig. 3f). Specifically, MTOR S1261 was also significantly expressed in the tumor tissues in EDAC (t-test, FC (tumor/normal) = 5.3, *p* = 0.019), which showed significantly positive association (Pearson correlation, R = 0.96, *p* = 2.4E-3) (Fig. 3g, h). Collectively, this study presented comprehensive proteomic characterization of EESCC and EDAC at multi-omics level, and revealed the functional pathways of cell cycle in EESCC, the positive impacts of apoptosis, and metabolic processes in EDAC, and further demonstrated the potential co-functions of RB1 and EGFR in ESCC, and the key role of MTOR at the protein and phosphoprotein levels in EDAC.

### Immune-based features of EESCC and EDAC

**Fig. 4:**
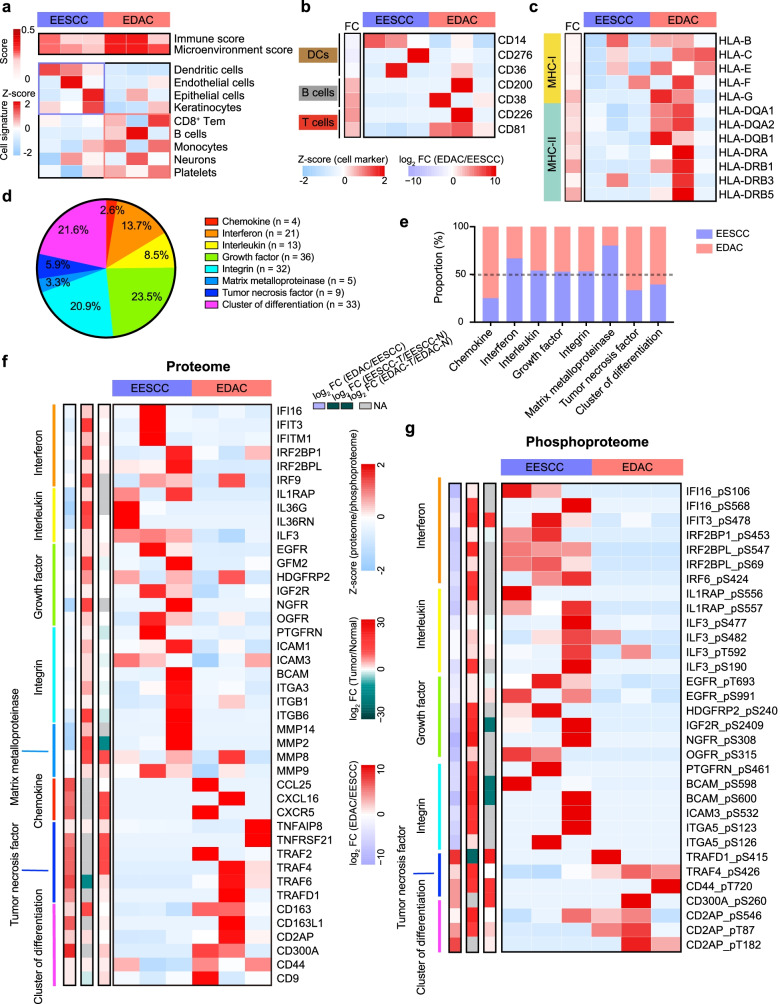
Immune-based features of EESCC and EDAC. **a** Heatmap showing the immune infiltration of EESCC and EDAC at the protein level. Top: heatmap showing the immune and microenvironment score. Bottom: heatmap showing the cell signatures. **b** The proteome-level expression of the biomarkers of DCs, B cells, and T cells in EESCC and EDAC. **c** Expression of MHC-I (top) and MHC-II (bottom) in EESCC and EDAC at the protein level. **d** Pie chart showing eight major classifications of cytokines identified in early-stage gastrointestinal cancer. **e** Bar charts illustrating the proportion of eight major classifications of cytokines in ESSCC (purple) and EDAC (red). **f** Heatmap showing the expression of the proteins of the major cytokines in EESCC and EDAC at the protein level. **g** Heatmap showing the expression of the proteins of the major cytokines in EESCC and EDAC at the phosphoprotein level

Recent studies have well-established the connection between inflammatory and tumorigenesis, and have considered the inflammatory is an important risk factor for gastrointestinal cancer [32]. To gain insight into features of immune infiltration of early-stage gastrointestinal cancer, we analyzed the proteomic profiles of EESCC and EDAC, and deconvoluted immune, stromal, and microenvironment cell signature using xCell (https://xcell.ucsf.edu) [33]. As a results, we found the cellular characteristics of dendritic cells (DCs) was dominant in EESCC, evidenced by the highly expressed biomarkers at the protein level, such as cluster of differentiation 14 (CD14), CD276, and CD36 (Fig. 4a, b; Supplementary Table S3). Furthermore, the cell signatures of endothelial cells, epithelial cells, and keratinocytes were prominent in EESCC, implying the specific characteristics of the esophagus tissues. Specifically, higher immune score (t-test, FC (EDAC/EESCC) = 1.39, *p* = 0.04) and microenvironment score (t-test, FC (EDAC/EESCC) = 1.35, *p* = 0.03) were observed in EDAC, evidenced by the overrepresentation of cell signatures, such as CD8^+^ Tem, B cells, monocytes, neurons, and platelets (Fig. 4a). In addition, the cell markers of B cells (e.g., CD200 and CD38) and T cells (e.g., CD226 and CD81) were also overrepresented in EDAC (Fig. 4b). In EDAC, the molecules of major histocompatibility complex class I and II (MHC-I/II), were highly expressed, including HLA-B, HLA-C, HLA-E, HLA-DQA1, HLA-DQB1, HLA_DRA, HLA-DRB1, etc. (Fig. 4c).

The increased pro-inflammatory cytokines permit immune activity in disease [34]. To gain insight of the difference of early-stage gastrointestinal cancer, we performed cytokines-classification analysis of all identified proteins (*n* = 7870) in early-stage gastrointestinal cancer. As a result, we identified 153 cytokines, which were classified into 8 major cytokines types as following: chemokine (2.6%, *n* = 4), interferon (13.7%, *n* = 21), interleukin (8.5%, *n* = 13), growth factor (23.5%, *n* = 36), integrin (20.9%, *n* = 32), matrix metalloproteinase (MMP) (3.3%, *n* = 5), tumor necrosis factor (5.9%, *n* = 9), and CD (21.6%, *n* = 33) (Fig. 4d). Comparative analysis elucidated proteins of chemokines, tumor necrosis factor, and CD were prevalent in EDAC, while the proteins of interferon, interleukin, growth factor, integrin, and MMP were prominent in EESCC (Fig. 4e and Supplementary Fig. S4). Specifically, the expression of IFI16 (S106 and S568), IFIT3 (S478), IRF2BP1 (S453), IRF2BPL (S69 and S547), IL1RAP (S556 and S557), ILF3 (S482 and T592), EGFR (T693 and S991), IGF2R (S2409), ICAM3 (S532), ITGA5 (S123 and S126), TRAF4 (S426), etc. was overrepresented in EESCC at the protein and phosphoprotein levels (Fig. 4f, g). In addition, we found those proteins/phosphoproteins were overrepresented in the tumor tissues of ESCC compared with paired normal tissues. In EDAC, the overrepresentation of the proteome expression and phosphorylation of CD44 (T720), CD300A (S260), and CD2AP (T87 and S458), was detected, which was also notably observed in the tumor tissues compared with paired normal tissues of EDAC. Taken together, we elucidated the higher immune infiltration in EDAC, disclosed the cytokines classification, and revealed the specific immune features of EESCC and EDAC.

### Proteomic kinases profiles of EESCC and EDAC

**Fig. 5:**
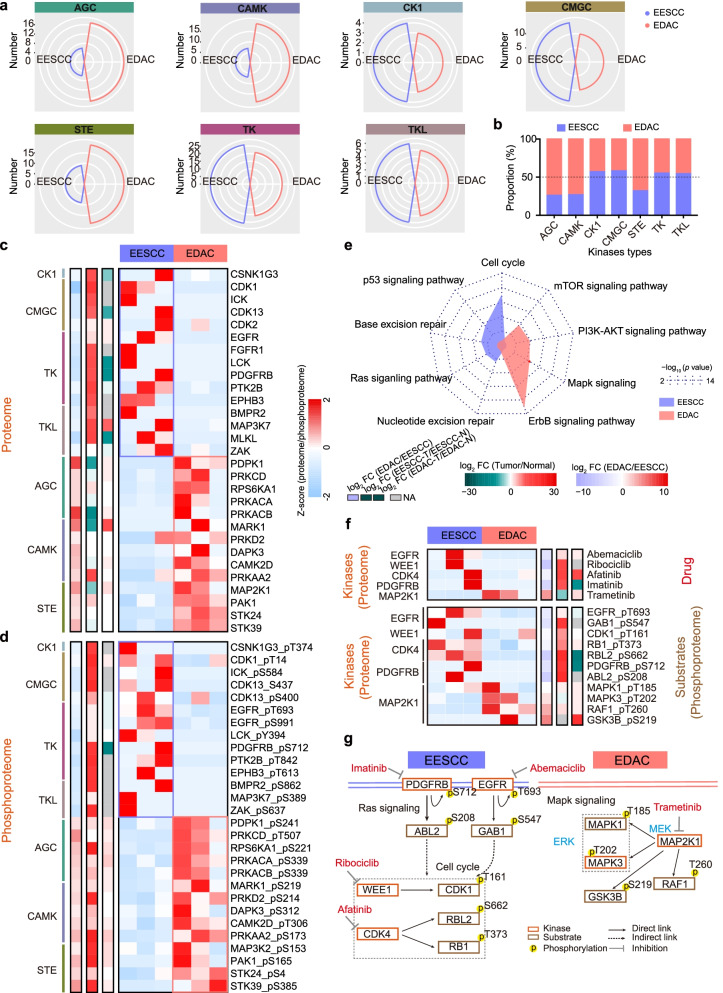
Proteomic kinases profiles in EESCC and EDAC. **a** Distribution and the number of the seven major kinases types in EESCC (purple) and EDAC (red). **b** Bar charts showing the differential proportion of major kinases types in EESCC (purple) and EDAC (red). **c** Heatmap representing the expression of the proteins of major kinases types in EESCC and EDAC at the protein level. **d** Heatmap representing the expression of the proteins of major kinases types in EESCC and EDAC at the phosphoprotein level. **e** Enrichment of the kinases and the downstream substates showing the dominant pathways in EESCC (purple) and EDAC (red). *P* value < 0.05 and FDR q value < 0.3 were considered as significant enrichment. **f** Heatmap showing the expression of the inhibitors (FDA drug) to kinases (top) (proteome) and the kinases regulated substates (phosphoproteome) in EESCC and EDAC. **g** Pathways based on the selected phospho-substates and kinases, with relevant drugs shown by targets in EESCC (left) and EDAC (right)

Human protein kinases mediate the majority of signal transduction pathways in biological processes, including cell metabolism, cell cycle, apoptosis, etc. [35], and the novel targets and inhibitors are applied to clinic strategy [36, 37]. To access the specific kinases of early-stage gastrointestinal cancer, the identified protein kinases (*n* = 166) were mapped to the human protein kinases. As a result, we depicted a Kinome Tree (Supplementary Fig. S5a), and found the more kinases of casein kinase 1 (CK1) group (57.1%), cyclin-dependent/mitogen-activated protein kinase (CMGC) group (58.3%), tyrosine-protein kinase (/receptor) (TK) group (55.3%), and Serine/Threonine-protein kinase (/receptor) (TKL) (54.5%) were detected in EESCC (Fig. 5a and Supplementary Fig. S5b). In addition, the kinases of cAMP/cGMP/calcium/phospholipid-dependent kinase (AGC) (73.9%), serine/tyrosine/threonine protein kinase (STE) group (73.1%), and Ca2+/CaM-dependent protein kinase (CAMK) group (67.9%) were overrepresented in EDAC (Fig. 5b).

Specifically, CSNK1G3, ICK, CDK1, CDK2, EGFR, FGFR1, PDGFRB, EPHB3, MAP3K7, ZAK, etc. were prominent in EESCC at the protein level. Consistently, the phosphorylation of CSNK1G3 T374, CDK1 T14, ICK S584, EGFR T693 and S991, LCK Y394, PDGFRB S712, EPHB3 T613, MAP3K7 S389 and ZAK S637, were overrepresented in EESCC at the phosphoprotein level (Fig. 5c, d). Comparably, those specific proteins/phosphoproteins were also highly expressed in the tumor tissues compared with paired normal tissues of EESCC. In EDAC, higher expressions of the proteome and phosphoproteome of PDPK1 (S241), PRKCD (T507), RPS6KA1 (S221), PRKACA (S339), MARK1 (S219), DAPK2 (S229), CAMK2D (T306), MAP3K2 (S153), PAK1 (S165), etc. were identified in EDAC. Consistently, those higher proteome−/phosphoproteome levels were also detected in the tumor tissues than paired normal tissues of EDAC.

To explore the functions of those kinase, we performed kinases-substates relationship analysis on the basis of kinases-substrates database [14, 16], and integrated the substrates in EESCC (*n* = 249) and EDAC (*n* = 297) (Supplementary Table S4). GO-enrichment analysis of those substates revealed the elevation of cell cycle (*p* = 2.9E-10), p53 signaling pathways (*p* = 5.2E-5), base excision repair (*p* = 4.6E-4), nucleotide excision repair (*p* = 2.4E-3), and Ras signaling pathways (*p* = 1.9E-3) in EESCC, and disclosed the overrepresentation of mTOR signaling (*p* = 3.3E-5), PI3K-AKT signaling pathways (*p* = 1.4E-5), Mapk signaling pathways (*p* = 2.6E-6), ErbB signaling pathways (*p* = 2.1E-13) in EDAC (Fig. 5e).

Kinases were applicated to the clinic strategy, such as anti-EGFR (abemaciclib) in breast cancer [38], and anti-MAP2K1 (trametinib) in melanoma [39]. We then accessed the drug targets approved by the US Food and Drug Administration database (FDA) (https://www.fda.gov) [40]. To an end, we found 6 kinases in our study were recorded in FDA datasets, in which EGFR, WEE1, CDK4, and PDGFRB were prominent in EESCC, and MAP2K1 was prevalent in EDAC (Fig. 5f). Phosphorylation impacts multiple cellular processes, with site occupancy tightly regulated by the activity of kinases and phosphatases [41]. We then performed integrative analysis of the differential kinases-substates (site), and proposed the functions of drugs approved by FDA in EESCC and EDAC. In EESCC, anti-EGFR with abemaciclib decreased the expression of EGFR (T693) and the downstream phosphorylation of GAB1 (S547), and anti-PDGFRB with imatinib in-activated the phosphorylation of PDGFRB (S712) and ABL (S210), which participated in cell cycle. Additionally, the inhibitor of ribociclib to WEE1 down-regulated the CDK1 at the protein and phosphoprotein levels, and the anti-CDK4 with afatinib decreased the phosphorylation of RBL2 (S662) and RB1 (T373), resulting in the stability of cell cycle-checkpoint which was the final safeguard of genomic fidelity (Fig. 5g). In EDAC, the inhibitor of trametinib to MAP2K1 was negative associated with the phosphorylation of MAPK1 (T185) and MAPK3 (T202), which down-regulated Mapk signaling and inhibited cell proliferation in EDAC (Fig. 5g). Collectively, we revealed EESCC-specific and EDAC-specific kinases, elucidated the functional kinases-substates relationship network, and proposed the potential clinic strategy in EESCC and EDAC, providing a novel insight for gastrointestinal cancer in the clinic.

## Discussion

Early screening and diagnosis provided better outcomes for patients, and are employed to many cancers, especially in gastrointestinal cancer. Great progress in mass spectrometry-based proteomics and advancement of FFPE samples enables to explore the molecular characterization of cancers, including CRC [10] gastric cancer [11] breast cancer [24] and so on. Whereas, the trace early-stage cancer sample is still a challenge, and the proteomic profiling of trace FFPE samples of early-stage cancer remain largely unknow.

In this study, we a performed comprehensive proteomic landscape of early-stage gastrointestinal cancer (EESCC and EDAC), and identified ~ 6000 proteins and > 10,000 phosphosites in single trace FFPE samples, and presented highly coverage at the protein and phosphoprotein levels, providing proteomic datasets and phosphoproteomic datasets of early-stage cancer. Comparative analysis revealed the difference between EESCC and EDAC was derived from their origin normal tissue, which allowed us to investigate the diverse proteomic characterization of EESCC and EDAC.

The distinct separation of EESCC and EDAC indicated the tumor heterogeneity and difference among cancer types in gastrointestinal cancer. We then performed SAM [27] analysis of EESCC and EDAC, and found that primary functions of normal esophagus tissues were prominent in EESCC, such as keratinization (e.g., KRT2, KRT5, etc.), epidermis development (e.g., CDH3, TCHH, etc.) at the protein and phosphoprotein levels. EGFR and RB1, played key roles in cell cycle, functioned in the metastasis and carcinogenesis of head and neck cancer and lung cancer [42, 43]. In EESCC, we found the high phosphorylation of EGFR T693 and RB1 T373, which showed significantly positive correlation, indicating the co-functions of EGFR and RB1 in the ESCC carcinogenesis at the phosphoprotein level. In EDAC, the high expression of metabolic proteins (e.g., ACO2, ATP5B, PFKL, etc.) and Mapk signaling pathways (e.g., MAPK1, MAPK3, ARAF, etc.) was detected, which was evidenced by the overrepresented phosphorylation of their corresponding phosphoproteins. Previous studies have proved the prominent mutations of *MTOR* and functions of mTOR signaling in small bowel cancer [31]. In this study, the overrepresentation of MTOR (S1261) at protein and phosphoprotein levels in EDAC, demonstrated the functions of MTOR in the carcinogenesis in duodenum cancer.

Inflammasome signaling is an emerging pillar of innate immunity, and the inflammatory microenvironment promotes gastrointestinal cancer development and invasion [44, 45]. To investigate the immune infiltration of early-stage gastrointestinal cancer, we accessed to xCell and deconvoluted the cell signature of EESCC and EDAC, and found high immune infiltration in EDAC. The cell signatures of CD8^+^ Tem, B cells, monocytes, etc., were prominent in EDAC, evidenced by the overrepresentation of CD200, CD226, CD81, MHC-I (e.g., HLA-B, HLA-E, etc.) and MHC-II (e.g., HLA-DQA1, HLA-DRA, etc.) proteins. Cytokines played key roles in inflammatory disease. We then performed cytokines classification of all identified proteins in early-stage gastrointestinal cancer, and found the prominent of interferon, interleukin, growth factor, integrin, and MMP in EESCC, and the prevalent of chemokine, tumor necrosis factor, and CDs in EDAC both at the protein and phosphoprotein levels.

Human protein kinases participated in the majority biological processes, including cell metabolism, cell cycle, apoptosis, immune system, etc. [35], and ubiquitous in tumors, such as lung cancer, breast cancer [46, 47]. Nowadays, kinases have become an important therapeutic target for the treatment, and inhibitors that target the kinases have been developed and are clinically active [48]. In this study, we depicted Kinome Tree of EESCC and EDAC, and found the kinases of CK1 groups, CMGC groups, TK groups, and TKL groups were overrepresented in EESCC, and the kinases of AGC group, CAMK group, and STE group were prevalent in EDAC at the protein and phosphoprotein levels. In addition, the kinases-substrates correlation network revealed the positive impacts of cell cycle, p53 signaling pathways, and DNA repair in EESCC, and the positive impacts of PI3K-AKT signaling pathways, mTOR signaling pathways, Mapk signaling pathways in EDAC. We also proposed the potential functional mechanism of drugs approved by FDA in EESCC and EDAC. For example, the inhibitor of trametinib to MAP2K1 decreased the phosphorylation of MAPK1 (T185) and MAPK3 (T202), which down-regulated Mapk signaling and inhibited cell proliferation in EDAC. However, limits still existed in our study. The more samples were need, and the trace amount samples restricted to collected genomic and transcriptomic data, and so on.

## Conclusion

This study presented a comprehensive proteomic landscape for early-stage gastrointestinal cancer for the first time, with identification of ~ 6000 proteins and > 10,000 phosphosites in single trace FFPE sample. We revealed the functional classification of all identified proteins and phosphoproteins in EESCC and EDAC, and found the diversity of the EESCC and EDAC was derived from their origin normal tissues. We also disclosed the distinct separation between EESCC and EDAC, and illustrating the impacts of cell cycle in EESCC, and of apoptosis and metabolic processes in EDAC at the protein and phosphoprotein levels. In addition, we deconvoluted the immune infiltration of early-stage gastrointestinal cancer, and found higher immune cell signatures in EDAC. Additionally, we revealed the specific cytokines in EESCC and EDAC. Furthermore, we delineated the Kinome Tree of EESCC and EDAC, elucidated the specific kinases, and proposed the potential clinic strategy in EESCC and EDAC, delivering a novel insight of the clinic therapeutic strategy for gastrointestinal cancer.

## Supplementary Information

**Additional file 1: Supplementary Fig. S1.** Overview of the proteomic profiles of EESCC and EDAC. **a** Schematic illustration of the samples and experimental workflow. **b** The number of the patient cases, identified proteins, and phosphosites in EESCC and EDAC. **c** Venn diagram showing the overlaps of the identified proteins (left) and phosphosites (right) in 3 EESCC patients. **d** Venn diagram showing the overlaps of the identified proteins (left) and phosphosites (right) in 3 EDAC patients. **Supplementary Fig. S2.** The phosphoproteomic features of tumor tissues compared with paired normal tissues in in EESCC and EDAC. **a** PCA analysis showing distinct separation between the tumor tissues and paired normal tissues in EESCC at the phosphoprotein level. **b** Volcano analysis depicted the differential expressed proteins of the tumor tissues and paired normal tissues in EESCC at the phosphoprotein level. **c** Bar chart presenting the functional pathways in up-regulated and down-regulated pathways in EESCC at the phosphoprotein level. **d** PCA analysis showing distinct separation between the tumor tissues and paired normal tissues in EDAC at the phosphoprotein level. **e** Volcano analysis depicted the differential expressed proteins of the tumor tissues and paired normal tissues in EDAC at the phosphoprotein level. **f** Bar chart presenting the functional pathways in up-regulated and down-regulated pathways in EDAC at the phosphoprotein level. **Supplementary Fig. S3.** Phosphoproteomic characteristics of EESCC and EDAC phosphoproteome. **a** Venn diagram representing the overlaps of the identification (left) and Pearson’s correlation coefficients (right) between proteome and phosphoproteome in EESCC. **b** Venn diagram representing the overlaps of the identification (left) and Pearson’s correlation coefficients (right) between proteome and phosphoproteome in EDAC. **c** PCA analysis showing distinct separation between normal tissues of EESCC and EDAC at the protein (left) and phosphoprotein (right) levels. **d** Volcano analysis depicted the differential expressed proteins of EESCC and EDAC. **e** Volcano analysis depicted the differential phosphorylation (left) and bar chart showing the functional pathways (right) in EESCC and EDAC phosphoproteome. **f** Phosphoproteins in functional pathways that were differentially expressed in EESCC (left) and EDAC (right) at protein and phosphoprotein levels. **g** Pearson’s correlation coefficients indicated significantly positive association between RB1 (T3730) and EGFR (T693). **Supplementary Fig. S4.** Eight major classifications of cytokines identified in early-stage gastrointestinal cancer. All the classified types (left) and the proteins of the cytokines identified in EESCC (middle) and EDAC (right) were shown. **Supplementary Fig. S5.** Major kinases in EESCC and EDAC. **a** A Kinome Tree of EESCC (purple) and EDAC (red) mapped to human kinases datasets. **b** The distribution of the major kinases types in EESCC (left) and EDAC (right).

**Additional file 2: Supplementary Table S1.** Matrix of identified proteins in EESCC and EDAC.

**Additional file 3: Supplementary Table S2.** Matrix of identified phophoproteins in EESCC and EDAC.

**Additional file 4: Supplementary Table 5.** The cell signatures of EESCC and EDAC.

**Additional file 5: Supplementary Table 6.** List of kinases and substrates in EESCC and EDAC.

## Data Availability

All the proteomic/phosphoproteomic raw data and the results files of EESCC and EDAC had been uploaded to the iProx Consortium (https://www.iprox.org/) with the PXD identifiers (PXD026736).
