# Supplementary material for: Integrative proteomic characterization of trace FFPE samples in early-stage gastrointestinal cancer
Source: Proteome Sci. 2022 Apr 9;20:5. doi: 10.1186/s12953-022-00188-0 (PMC8994365; doi:10.1186/s12953-022-00188-0)

Supplementary Fig. S1

a

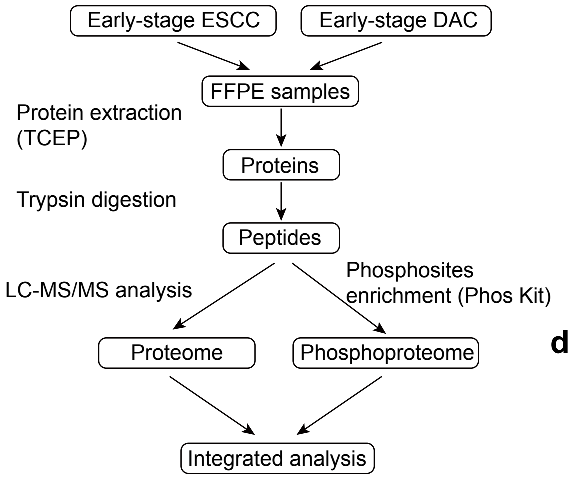

b

Cancer types	Cases	Proteins	Phosphosites
EESCC	3	4,667 (Normal)	6,192 (Normal)
		6,450 (Tumor)	18,072 (Tumor)
EDAC	3	6,562 (Normal)	10,022 (Normal)
		6,789 (Tumor)	12,200 (Tumor)

c

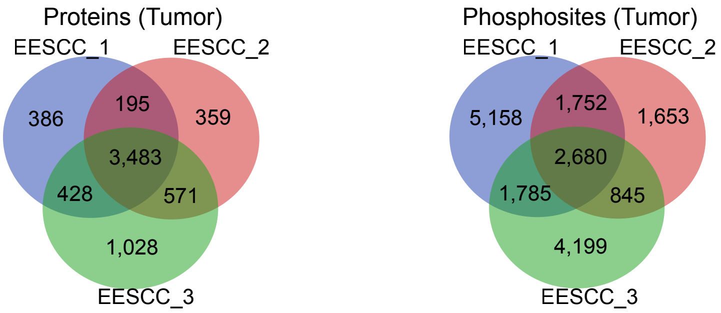

d

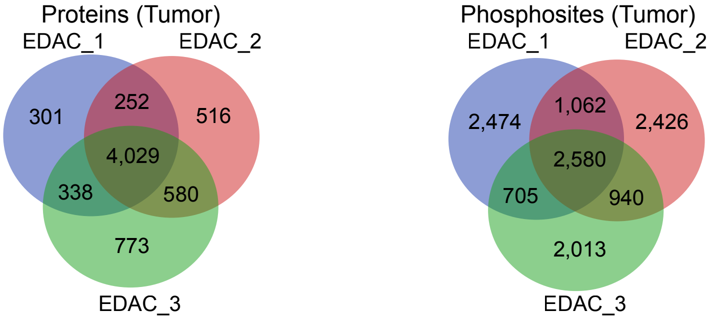

Supplementary Fig. S2

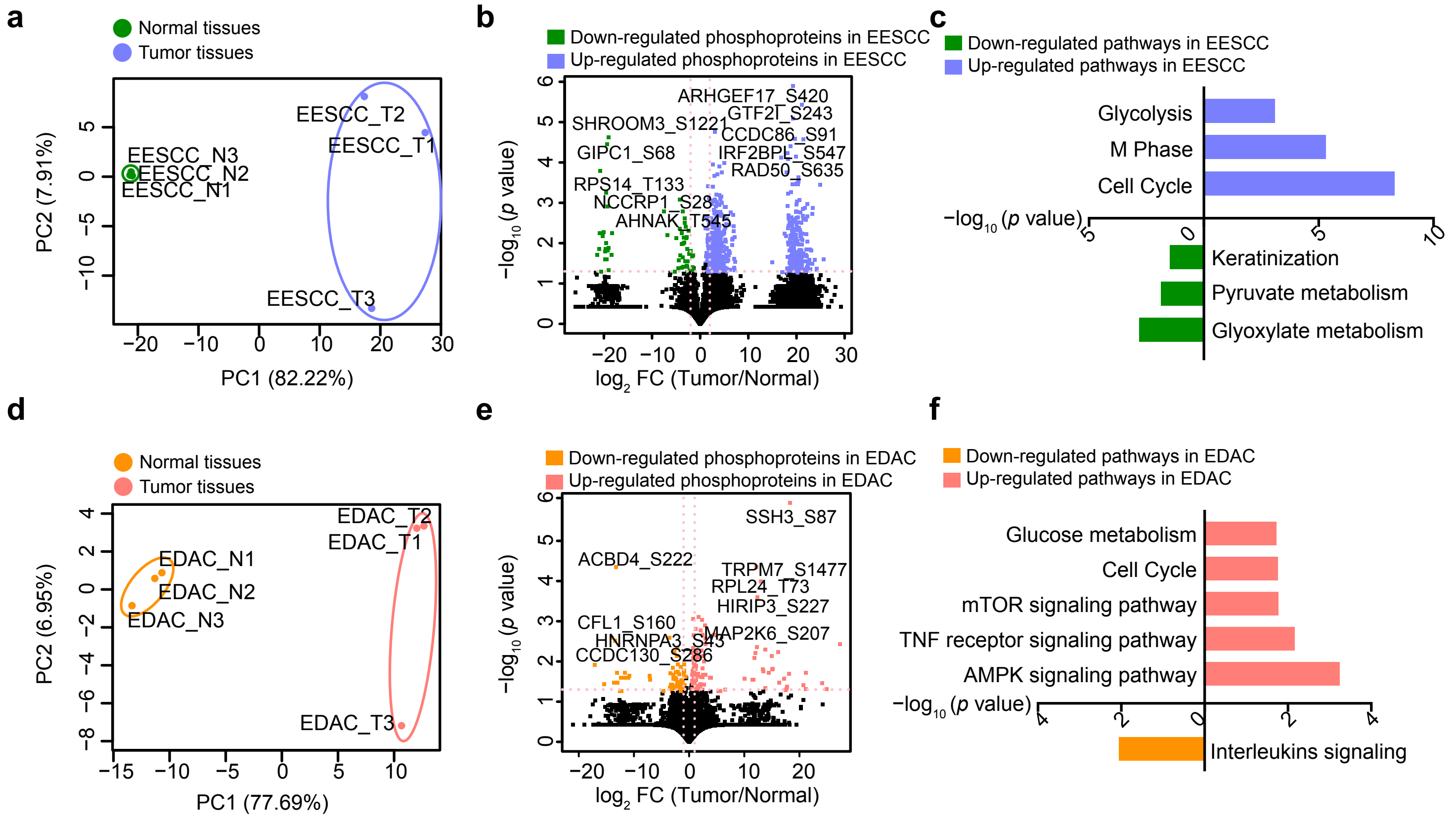

Supplementary Fig. S3

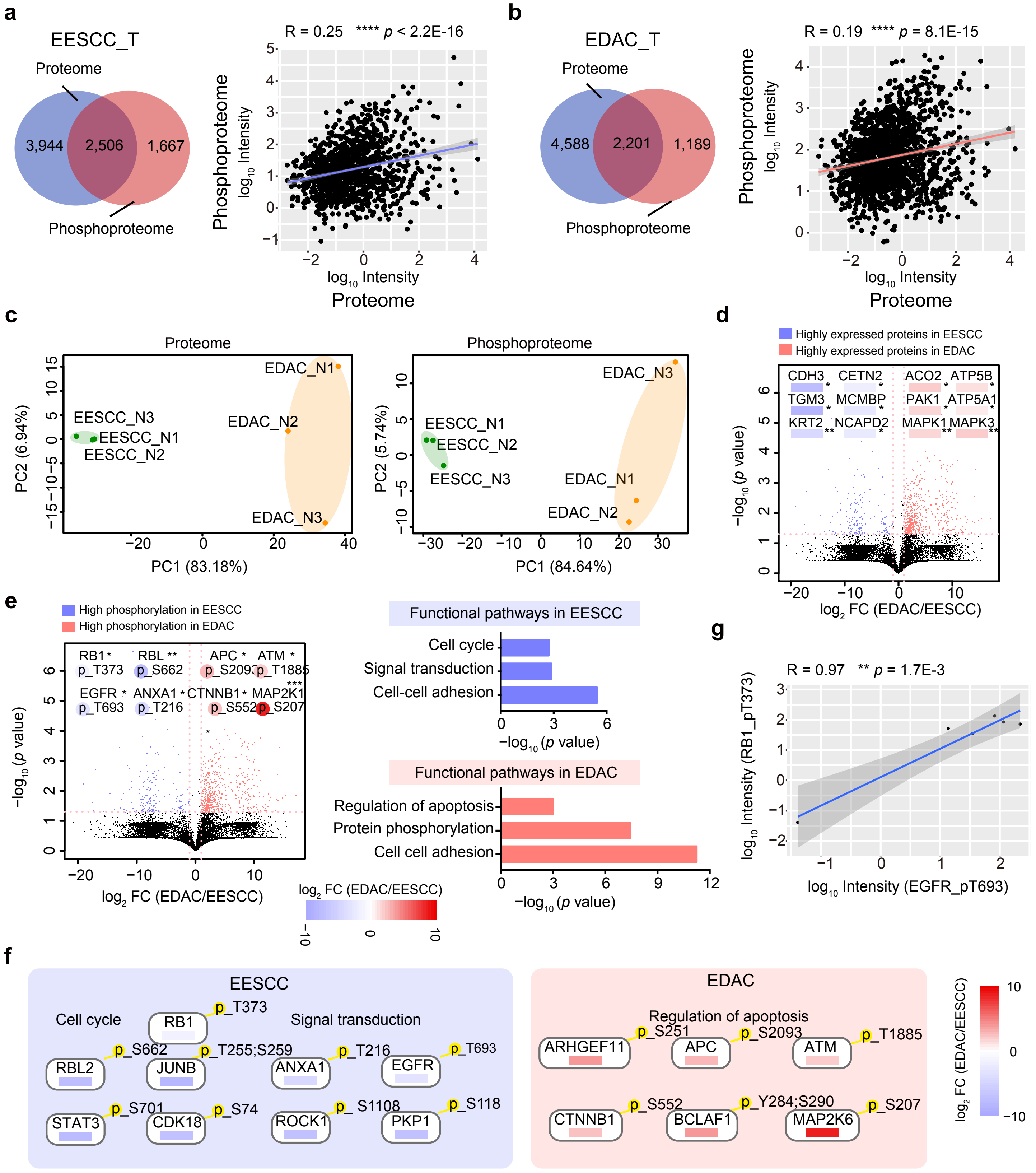

Supplementary Fig. S4

Cytokines	EESCC	EDAC
Chemokine (n = 4)	CCL19.	CCL25, CXCL16, CXCR5.
Interleukin (n = 13)	IL1RAP, IL1RN, IL36A, IL36G, IL36RN, ILF3, ILK.	IL16, IL18, IL4I1, ILF2, ILKAP, ILVBL.
Interferon (n = 21)	IFI16, IFI44, IFI44L, IFIH1, IFIT1, IFIT2, IFIT3, IFIT5, IFITM1, IFITM3, IRF2BP1, IRF2BPL, IRF6, IRF9.	IFI30, IFI35, IFITM2, IRF2BP2, IRF3, IRF4, IRF7.
Growth factor (n = 36)	EGFR, FGF19, FGF23, FGFR1, FGFR2, FGFR3, FGFR4, GFM2, HDGFRP2, HGF, IGF2BP1, IGF2BP3, IGF2R, IGFBP7, NGFR, OGFR, PDGFRB, PTGFRN, TGB1.	AGFG1, AGFG2, FGF22, FGFR1OP, GFAP, GFER, GFM1, GFPT1, GFPT2, HDGF, HDGFRP3, IGF1R, IGF2BP2, IGFALS, IGFBP2, TGFB1I1, TGFBI.
Integrin (n = 32)	ICAM1, ICAM2, ICAM3, ALCAM, BCAM, CAMP, VCAM1, ITGA2B, ITGA3, ITGA5, ITGAL, ITGAM, ITGAV, ITGAX, ITGB1, ITGB5, ITGB6.	L1CAM, MCAM, NCAM1, PECAM1, SCAMP1, SCAMP2, SCAMP3, ITGA1, ITGA2, ITGA6, ITGA9, ITGB1BP2, ITGB2, ITGB3, ITGB4.
Matrix metalloproteinase (n = 5)	MMP14, MMP2, MMP8, MMP9.	MMP1.
Tumor necrosis factor (n = 9)	TNFAIP2, TRAF3IP2, TRADD.	TNFAIP8, TNFRSF21, TRAF2, TRAF4, TRAF6, TRAFD1.
Cluster of differentiation (n = 33)	CD14, CD276, CD36, CD109, CD207, CD248, CD2BP2, CD3E, CD47, CD55, CD70, CD74, CD82.	CD200, CD226, CD38, CD81, CD163, CD163L1, CD209, CD2AP, CD300A, CD3EAP, CD44, CD48, CD59, CD5L, CD68, CD9, CD93, CD97, CD99L2, CDA.

Supplementary Fig. S5

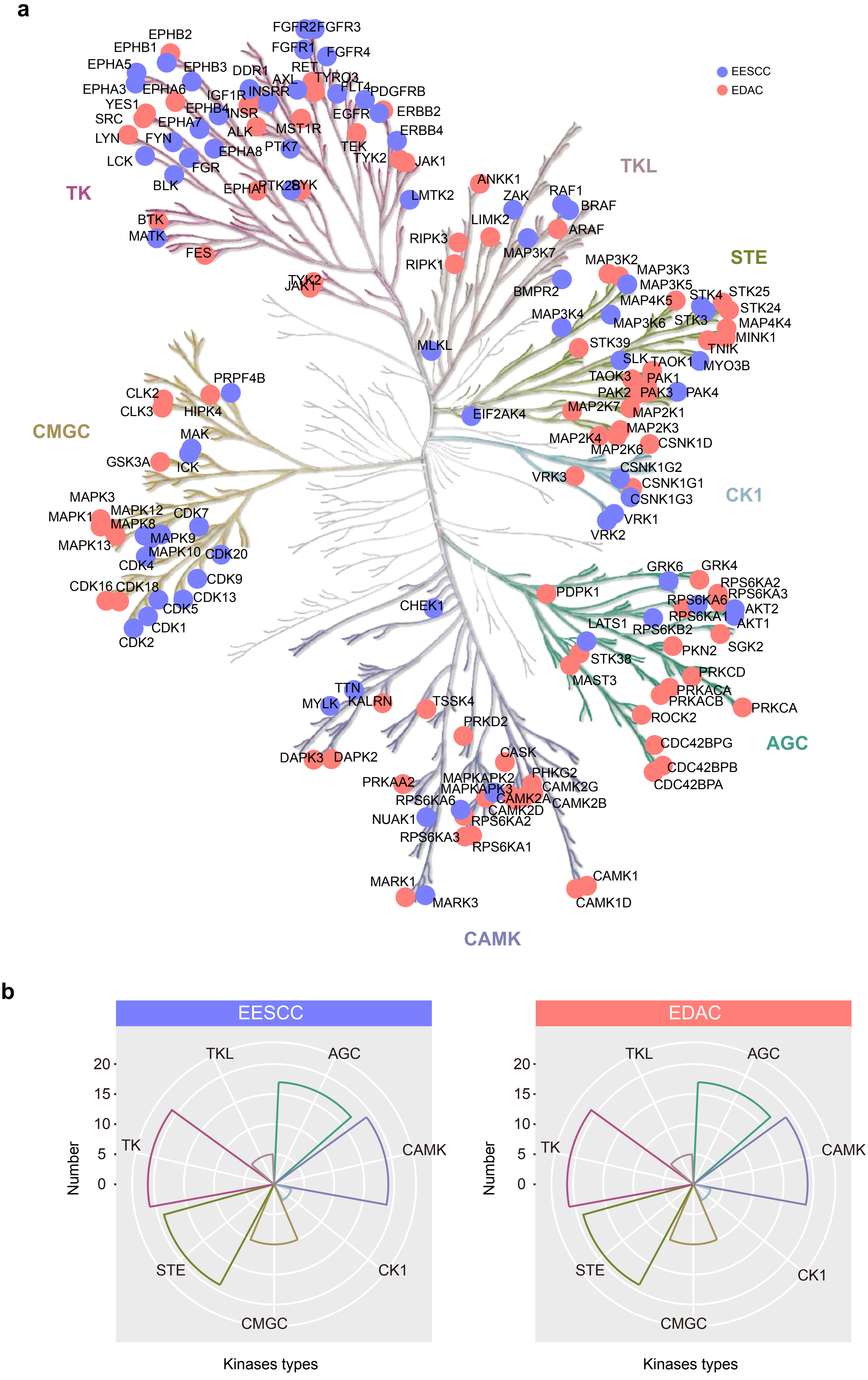

Supplement: Supplementary file 1 — Additional file 1: Supplementary Fig. S1. Overview of the proteomic profiles of EESCC and EDAC. a Schematic illustration of the samples and experimental workflow. b The number of the patient cases, identified proteins, and phosphosites in EESCC and EDAC. c Venn diagram showing the overlaps of the identified proteins (left) and phosphosites (right) in 3 EESCC patients. d Venn diagram showing the overlaps of the identified proteins (left) and phosphosites (right) in 3 EDAC patients. Supplementary Fig. S2. The phosphoproteomic features of tumor tissues compared with paired normal tissues in in EESCC and EDAC. a PCA analysis showing distinct separation between the tumor tissues and paired normal tissues in EESCC at the phosphoprotein level. b Volcano analysis depicted the differential expressed proteins of the tumor tissues and paired normal tissues in EESCC at the phosphoprotein level. c Bar chart presenting the functional pathways in up-regulated and down-regulated pathways in EESCC at the phosphoprotein level. d PCA analysis showing distinct separation between the tumor tissues and paired normal tissues in EDAC at the phosphoprotein level. e Volcano analysis depicted the differential expressed proteins of the tumor tissues and paired normal tissues in EDAC at the phosphoprotein level. f Bar chart presenting the functional pathways in up-regulated and down-regulated pathways in EDAC at the phosphoprotein level. Supplementary Fig. S3. Phosphoproteomic characteristics of EESCC and EDAC phosphoproteome. a Venn diagram representing the overlaps of the identification (left) and Pearson’s correlation coefficients (right) between proteome and phosphoproteome in EESCC. b Venn diagram representing the overlaps of the identification (left) and Pearson’s correlation coefficients (right) between proteome and phosphoproteome in EDAC. c PCA analysis showing distinct separation between normal tissues of EESCC and EDAC at the protein (left) and phosphoprotein (right) levels. d Volcan [file 12953_2022_188_MOESM1_ESM.pdf]
